# Copy number variations in Saudi family with intellectual disability and epilepsy

**DOI:** 10.1186/s12864-016-3091-6

**Published:** 2016-10-17

**Authors:** Muhammad I. Naseer, Adeel G. Chaudhary, Mahmood Rasool, Gauthaman Kalamegam, Fai T. Ashgan, Mourad Assidi, Farid Ahmed, Shakeel A. Ansari, Syed Kashif Zaidi, Mohammed M. Jan, Mohammad H. Al-Qahtani

**Affiliations:** 1 Center of Excellence in Genomic Medicine Research, King Abdulaziz University, Jeddah, 21589 Saudi Arabia; 2 Department of Pediatrics, Faculty of Medicine, King Abdulaziz University, Box 80215, Jeddah, 21589 Saudi Arabia

**Keywords:** Epilepsy, CNVs, Intellectual disability, Array-CGH, Saudi population

## Abstract

**Background:**

Epilepsy is genetically complex but common brain disorder of the world affecting millions of people with almost of all age groups. Novel Copy number variations (CNVs) are considered as important reason for the numerous neurodevelopmental disorders along with intellectual disability and epilepsy. DNA array based studies contribute to explain a more severe clinical presentation of the disease but interoperation of many detected CNVs are still challenging.

**Results:**

In order to study novel CNVs with epilepsy related genes in Saudi family with six affected and two normal individuals with several forms of epileptic seizures, intellectual disability (ID), and minor dysmorphism, we performed the high density whole genome Agilent sure print G3 Hmn CGH 2x 400 K array-CGH chips analysis. Our results showed *de novo* deletions, duplications and deletion plus duplication on differential chromosomal regions in the affected individuals that were not shown in the normal fathe and normal kids by using Agilent CytoGenomics 3.0.6.6 softwear. Copy number gain were observed in the chromosome 1, 16 and 22 with *LCE3C*, *HPR*, *GSTT2*, *GSTTP2*, *DDT* and *DDTL* genes respectively whereas the deletions observed in the chromosomal regions 8p23-p21 (4303127–4337759) and the potential gene in this region is *CSMD1* (OMIM: 612279). Moreover, the array CGH results deletions and duplication were also validated by using primer design of deleted regions utilizing the flanked SNPs using simple PCR and also by using quantitative real time PCR.

**Conclusions:**

We found some of the *de novo* deletions and duplication in our study in Saudi family with intellectual disability and epilepsy. Our results suggest that array-CGH should be used as a first line of genetic test for epilepsy except there is a strong indication for a monogenic syndrome. The advanced high through put array-CGH technique used in this study aim to collect the data base and to identify new mechanisms describing epileptic disorder, may help to improve the clinical management of individual cases in decreasing the burden of epilepsy in Saudi Arabia.

## Background

Epilepsy in human is among one of the utmost common brain disorder with 1 % occurrence and characterized by regular and unprovoked seizures due to irregular electric activity in central nervous system (CNS) [1]. Until now there are more than 50 different epileptic syndromes with wide range of clinical features have been identified. Epilepsy can be divided as idiopathic or symptomatic epilepsies. Infections, metabolic disorders, brain tumors, head trauma, or stroke are the main reasons of symptomatic epilepsy whereas genetic contributions are the main cause of the idiopathic seizure [2]. In human genetics structural changes are largely unexplored area that may be the common factor of epileptic disease [3]. These changes include deletions, duplications insertions, inversions, and translocations of DNA sequences, and includes copy number differences also known as CNVs [4, 5]. Recently the development of advanced technologies for genome wide studied such as array-CGH helped to detect minute amplifications and deletion in the chromosomal region [6, 7]. CNVs are the most important component of genetic variation and act as important players in the genetic etiology of various neurodevelopmental disorders. CNVs are developing as an important genetic contribution to identify a wide range of epilepsies, such as common new discoveries in epilepsies from genetic generalized epilepsies to the individually rare particular epileptic of encephalopathies [8]. The role of CNVs highlighted in the etiology of number of disorders such as intellectual disability (ID) [9] autism [10, 11], schizophrenia [12, 13] and epilepsy [14, 15]. Now a days various free databases for pathogenic and normal genome variations are available on internet and these are exceptionally valuable tools for interpretation of CNVs identified in normal and patients such as Database of Chromosomal Imbalance and Phenotype in Humans Using Ensembl Resources: DECIPHER and Database of Genomic Variants: DGV;).

In this study, we observed *de novo* CNVs gain and deletion in a large family with affected mother and five affected kids having intellectual disability, epilepsy and some member with mild intellectual disability nd we hypothesized that genes found within those CNVs would be novel candidate for epilepsy. We are reporting *de novo* CNVs and the genes cluster in that deleted or amplified region only in the affected members of the family that might be helpful to understand the genetic etiology of epilepsy.

## Results and discussion

**Fig. 1:**
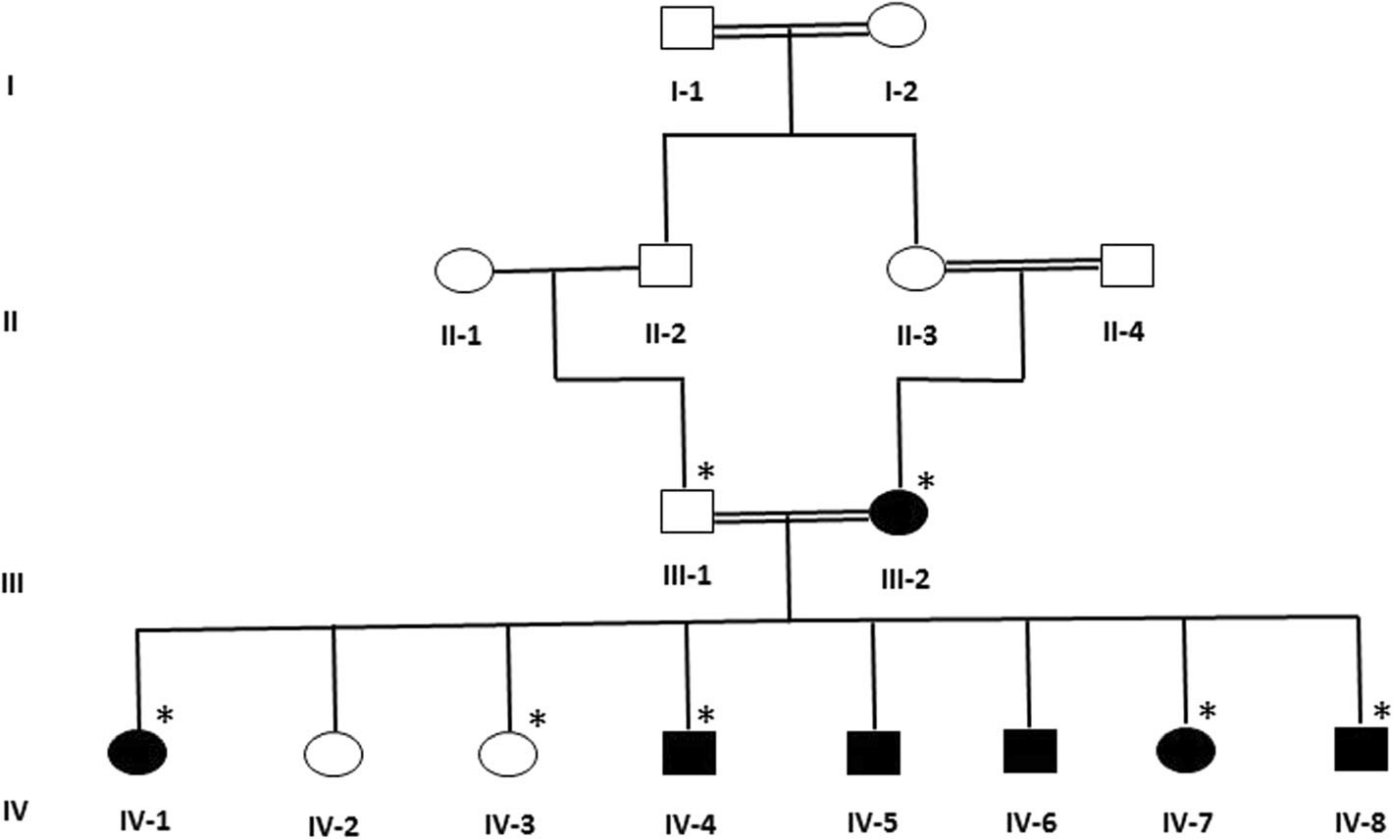
A consanguineous family pedigree from Saudi Arabia with intellectual disability and epilepsy. The available samples for microarray and validation study are marked with asterisks

Our Array-CGH results showed deletion as well as duplication in different genomic regions of effected member of the epileptic patients of the family (Fig. 1). In this data, we reported the results of the family with five affected member satisfying the cut off value of gain and deletions (−1.0 for deletion and 0.8 for duplication). Deletion was observed in chromosome 8 with cytoband 8p23-p21 *CSMD1* gene and copy number gain were observed in the chromosome 1, 16 and 22 with *LCE3C*, *HPR*, *GSTT2* and *GSTTP2* genes respectively. These observed CNV findings were confirmed by using the qPCR analysis.

### Microarray data showed deleted CNVs

**Fig. 2:**
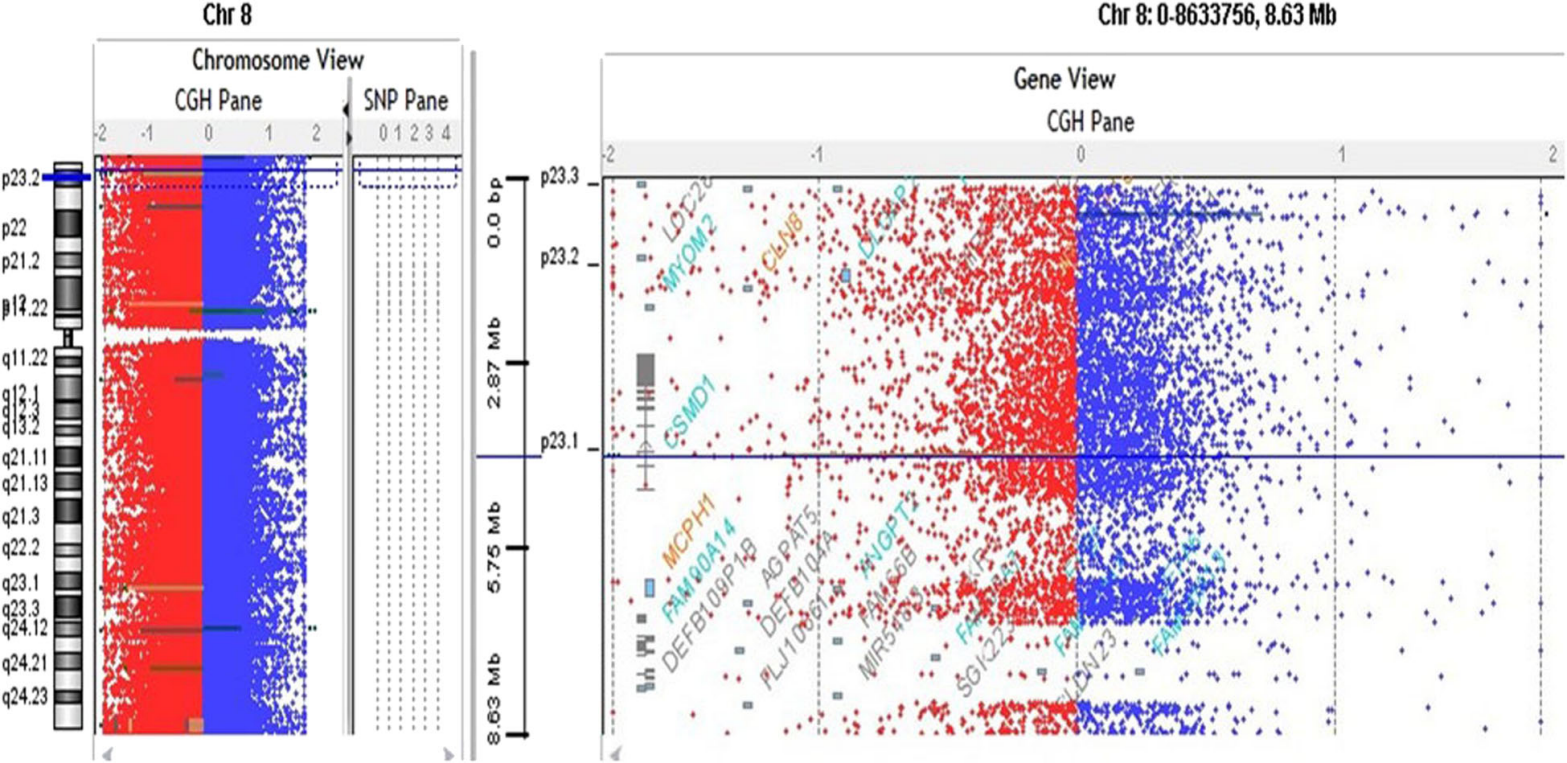
Array-CGH profiles analysis using Agilent CytoGenomic Analytics software (V.3.0.6.6) showing the deletion. Zero value indicates equal fluorescence intensity ratio between the sample and reference. Copy number losses shifted the ratio toward left (*red*), whereas copy number gains towards the right side (*blue*). CNV deletions observed in the chromosomal regions 8p23-p21 in four affected members of the family starts from 4,303,127 end 4,337,759 and the potential gene in this region is *CSMD1* (OMIM: 612279)

Our results of whole genome 2x 400 K oligonucleotide microarray analysis showed deletions 8p23.1 start 4,310,831 end 4,329,349 in four affected member of the family and important gene in this region name as CUB and sushi multiple domains 1 (*CSMD1*) (Fig. 2). This gene is large size (1 Mb) similar to CSMD3 which maps in the region of 8q23 and already been associated with mental retardation, autism and to epilepsy [16–19].

The gene CSMD1 is a unique multiple domain complement-regulatory protein, which is highly expressed in the epithelial tissues and CNS and play a role as important regulator of complement initiation and inflammation in the developing.

CNS and also play role in the context of growth cone function [20]. The deleted region in our patient is at the start of *CSMD1* and all the affected member having the language delay, learning difficulties and epilepsy as shown to be totally deleted (haploinsufficiency) and interrupted by the breakpoint patients with autism and ‘severe language delay’ and learning difficulties reported by [21, 22].

**Fig. 3:**
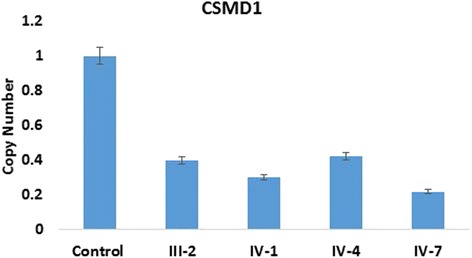
Confirmation of deleted and gained CNVs region by using qPCR analysis. The results have shown *CSMD1*gene copy number deletions in the mother and three affected member of the family showed significant fold change as compared to the control

These deletion were confirmed by qPCR which have shown a significant deletions in the gene copy number in affected mother and three kids he same family (Fig. 3). We screened ethnically matched healthy control chromosomes (*n* = 50) to ensure that the copy number deletions did not represent a normal population, and verified that it was not present outside the family.

### Microarray data showed CNVs copy number gain

**Fig. 4:**
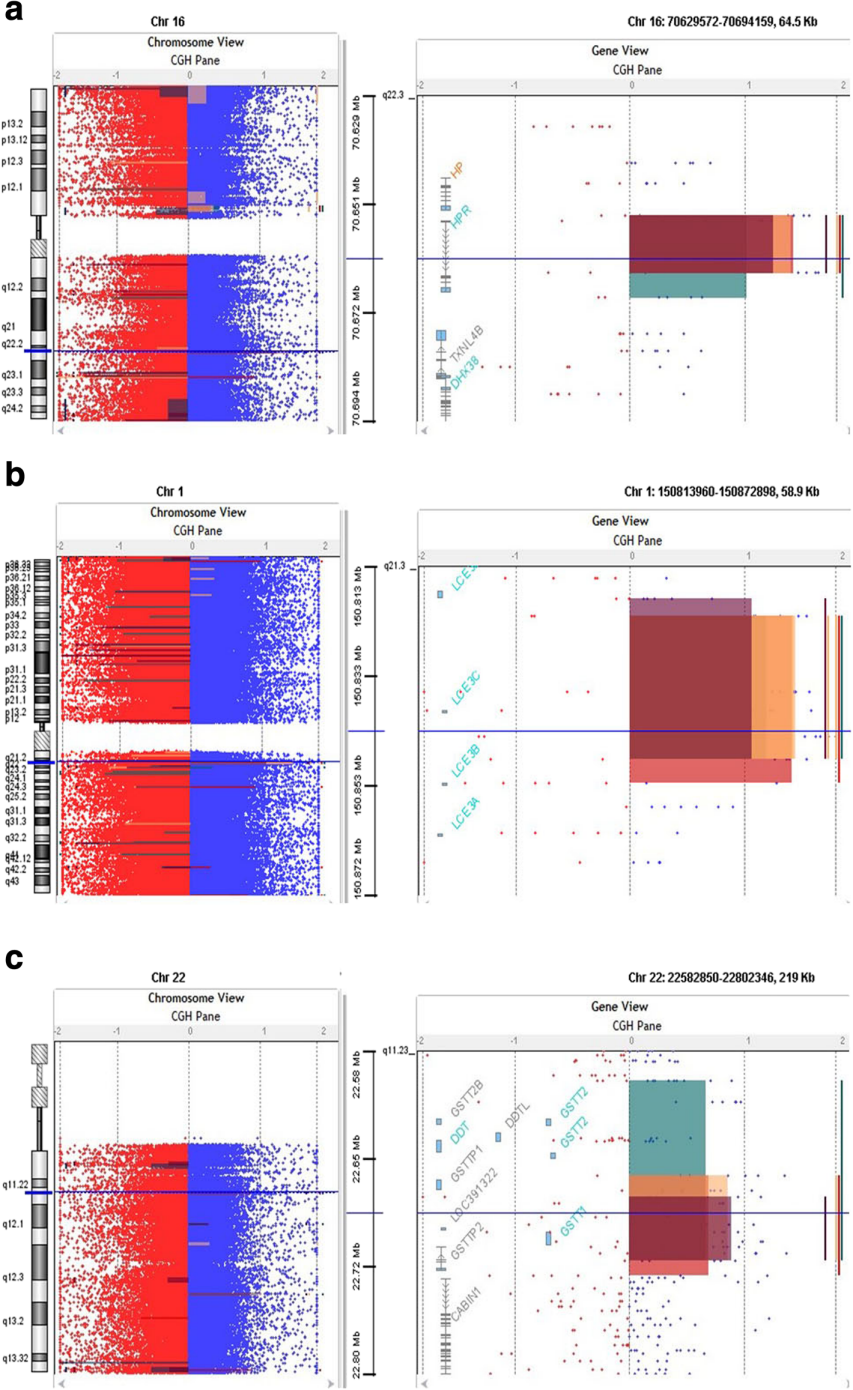
**a** Array-CGH profiles analysis using Agilent CytoGenomic Analytics software (V.3.0.6.6). Zero value indicates equal fluorescence intensity ratio between the sample and reference. Copy number losses shifted the ratio toward left (*red*) whereas copy number gains towards the right side (*blue*). CNVs gain at chromosome 1 starts from 150,819,879–150,819,938 cytoband 1q21.3 in the four affected members of the family and the potential gene in this region is *LCE3C*. **b** Our results showed the gain at chromosome 16 that starts from 70,647,078–70,669,681 cytoband 16q22.2 in our all four affected members of the family and the potential gene in this region is *HPR* gene. **c** Our results showed gain at chromosome 22 starts 22,686,690–22,735,300 cytoband 22q11.23 and the potential gene cluster in this region are *GSTT1*, *GSTTP2*, *GSTT2B*, *GSTT2*, *DDT*, and *DDTL* in all affected members of the family

Our results of whole genome 2x 400 K oligonucleotide showed CNVs gain at chromosome 1 in the four affected members of the family starts from 150,819,879–150,819,938 cytoband 1q21.3 and the potential gene in this region is *LCE3C* (Fig. 4). Previously *LCE* deletion was related with psoriasis [23, 24] but no region have been associated with epilepsy or autism. Recently, late comified envelope (*LCE*) genes especially the cluster 3 (*LCE3*) genes mutation influence developing psoriasis and psoriatic arthritis [25]. We found this CNV in our four affected individuals and this amplification was not seen in the normal individuals of the family. Further, in future analysis will be necessary to determine if this CNV especially the *LCE3C* gene is an epilepsy and autism risk variant in Saudi population (Fig. 4b).

We also found gain at chromosome 16 that starts from 70,647,078–70,669,681 cytoband 16q22.2 in our all five affected members of the family and the potential gene in this region is *HPR* gene code haptoglobin protein in plasma acts as a scavenger for free heme, and haptoglobin-related protein (coded by the *HPR* gene) (Fig. 4a). In African-Americans HPR gene copy number variation has been reported, where extra copies of the HPR gene have been produced by non-allelic homologous recombination [26].

Previously, duplication of *HPR* gene showed a slight, non-significant under transmission to human African trypanosomiasis-affected children from normal parents in the Democratic Republic of Congo [27]. There is no study available that link this gene to epilepsy, intellectual disability and autism so we suggest further investigation of this duplication the Saudi population.

Our micro array data showed another copy number gain at chromosome 22 starts 22,686,690–22,735,300 cytoband 22q11.23 and the gene cluster in this region is *GSTT1*, *GSTTP2*, *GSTT2B*, *GSTT2*, *DDT*, and *DDTL*. Additional analysis through qPCR confirmed the duplications that has shown a significant fold increase in the *GSTT1*, *GSTTP2*, *GSTT2B GSTT2* gene copy number compared to the controls (Fig. 4c). Glutathione S-transferase theta 1 (*GSTT1*) are a member of superfamily of enzymes that play role in both protection from oxidative damage and detoxification [28]. In liver they play an important role in metabolizing antiepileptic drugs (AEDs) [29, 30]. Many AEDs produce active metabolites, including epoxides, which might have result on suppression of epileptic spike, but inappropriately, also in systemic toxicity through covalent binding to lipids and proteins [29]. Glutathione S-transferase (GSTs) help in catalyzing the conjugation of these metabolites to glutathione, particularly in the elimination of epoxide metabolites that are produced during AEDs metabolism. GSTs not only help in decreasing their toxicity and helping their excretion from the body, it is expected that GSTs may also affect the response to anticonvulsant therapy [29, 31].

**Fig. 5:**
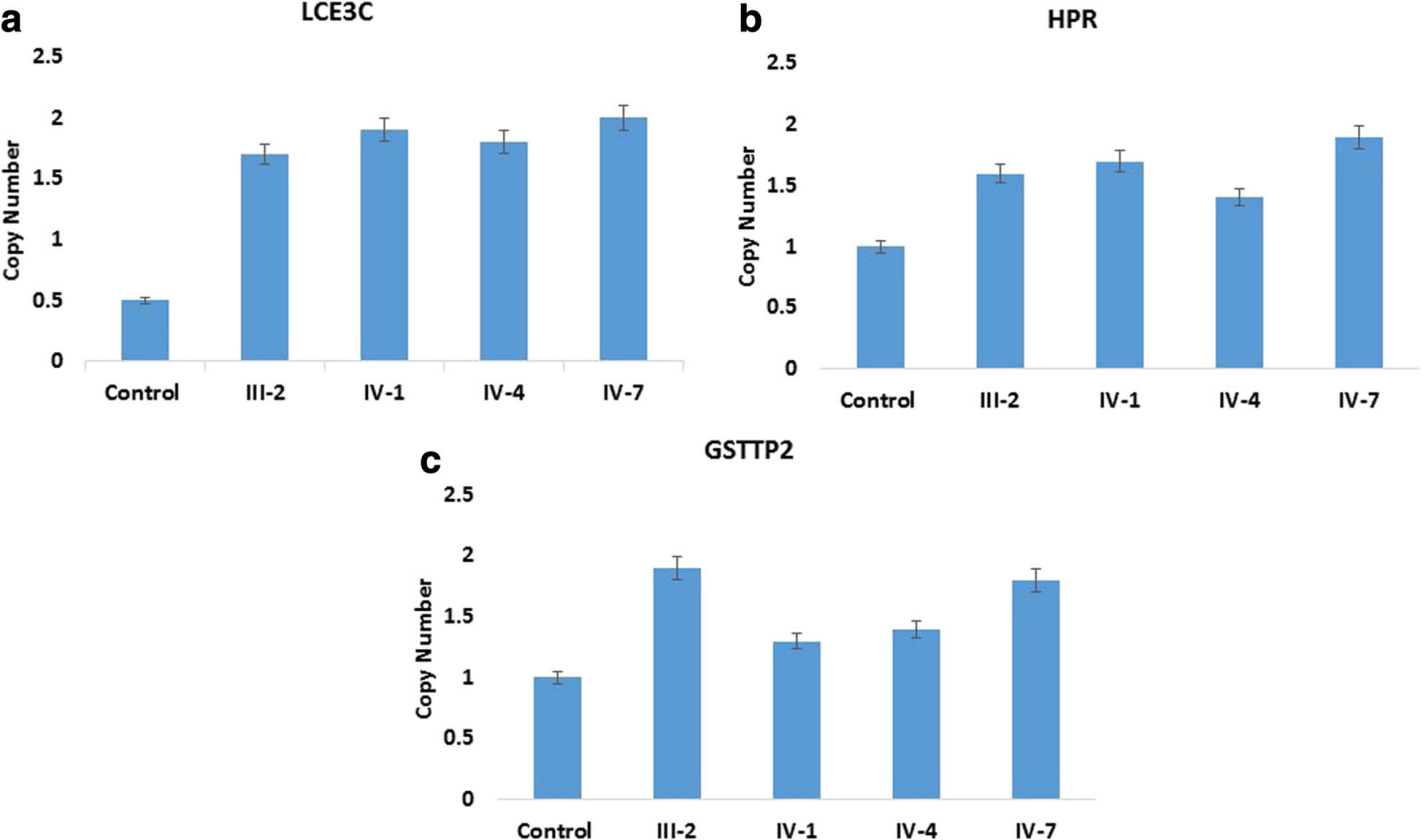
**a**, **b**, **c** Confirmation of CNVs by qPCR have shown a significant fold increase in **a** *LCE3C*, **b** *HPR*, and **c** *GSTT2* gene copy number in the patients of the family member as compared to the healthy individuals

Previously it has been reported that replication of the 22q11.23 region associated with schizophrenia [32–37] further support the hypothesis that effect of copy number of GSTT2 may be that the regional structural variation would impact the risk of schizophrenia by altering controlling elements for other genes close to the region. Actually, this area shows a high density of segmental duplications and CNV that may impact the expression of surrounding genes in that area [38, 39] Furthermore, the discovery of disease linked with *GST* genes elsewhere in the genome, suggests a direct effect of *GSTT2* gene changes leading to the risk of schizophrenia [40]. Our microarray data showing the similar information that this region is involved in epilepsy as previously explained [40] duplication at chromosome 22 with gene cluster *GSTT1*, *GSTTP2*, *GSTT2B*, *GSTT2*, *DDT* and *DDTL* showing the similar phenotypes in our clinical information in the affected member of the family with epilepsy, intellectual disability and also leading to schizophrenia may be the candidate genes for this complex syndrome (Fig. 4c). These duplications were confirmed by qPCR which have shown a significant increase in the gene copy number in only four patients of the same family (Fig. 5a, b, c).

## Conclusion

In the development of neurological disorders CNVs play important role and may contribute in the genetic etiology of epilepsy. Our results showed novel CNVs/genes for the first time in Saudi family with six affected member with intellectual disability and epilepsy. Our results enhanced the knowledge of the copy number variants underling in epilepsy in Saudi population. This could provide a basis for the understanding of the chromosomal region that will be critical in the genome may be involved in the development of epilepsy, intellectual disability and further linked with schizophrenia. Further detailed studies and complete clinical information of the patients and identification of possible causative CNVs and genes in the CNV regions using array technology will be obligatory to identify novel syndromes that will help to improve the diagnosis of epilepsy.

## Methods

### Epileptic family

A large consanguineous pedigree of epileptic’s family with six affected (two female and all four male affected) and two normal individuals along with normal father and affected mother was enrolled for this study as shown in Fig. 1. Written informed consent was obtained from the study participants themselves or from the parents of children. The study was approved by the Center of Excellence in Genomic Medicine and Research King Abdulaziz University, following Helsinki Declaration of research and ethics standards. The family was primarily diagnosed as epileptic family and all the children and mother having epilepsy along with different clinical features such as mother was diagnosed as juvenile myoclonic epilepsy as the seizure started at the age of 16 years whereas the proband IV-1 having epilepsy with myoclonic seizures, muscle twitching along with uncontrolled jerking of the body sickle cell anemia and osteoporosis, proband lV-4 having epilepsy with myoclonic seizures and mild intellectual disability, while proband IV-6 having seizure with muscle twitching and uncontrolled body jerk.with speaking problems and IV-7 and IV-8VIII having seizure with myoclonic jerks with intellectual disability. In all the affected member of the family the seizure was started at different age group such as in mother seizure started when she was 16 years old and still suffering with this disease.

### DNA preparation

All the family member visited the clinic and blood samples from affected and normal individuals has been taken at King Abdulaziz University Hospital with their informed consent after the approval of the ethical committee. Genomic DNA was obtained from the blood through the QIAamp DNA blood mini kit followed by the protocol from the manufacturer.

### Array-CGH analysis

#### Genomic DNA fragmentation

The array-CGH analysis was performed by Agilent sure print G3 Hmn CGH 2x 400 K arrays (Agilent Technologies, USA) followed the protocol as provided by the manufacturer. Briefly, 500 ng of patient’s DNA along reference DNA of the similar sex (Promega, USA). *RsaI* and *AluI* enzymes used to digest the DNA and incubated for 2 h at 37 °C. The reference DNA was heat fragmented for 10 min at 95 °C.

### Fluorescent labeling, purification and hybridization

The digested DNA from affected and normal family member were labeled via random priming through Agilent labeling kit (Agilent Technologies, USA) following the manufacturer guidelines. Patient’s and reference DNA was labelled with Cy5-dUTPand Cy3-dUTP respectively. For the purification of the labelled samples, the Microcon YM-30 filter units were used (Millipore, USA). The DNAs from patient and reference were mixed with Cot-1 DNA (Invitrogen, USA) used as blocking agent along with hybridization buffer as per manufacturer instructions. Hybridization was done at 65 °C for 40 h, after the denaturation at 95 °C.

### Microarray chip washing and scanning

After the hybridization, microarrays and gaskets were disassembled in wash buffer 1 (Agilent Technologies). The slides were shifted to the wash buffer 2 after 05–30 min, (Agilent Technologies) and agitated at 37 °C for 01 min followed by the washing of slides with anhydrous acetonitrile. Scanning and image analysis were performed as per oligonucleotide array-CGH protocol (Agilent, version 4.0). Microarrays were scanned through Agilent Scanner (G2505C) and the data was extracted through Agilent’s Feature Extraction software (V.1.5.1.0).

### Data analysis

Agilent CytoGenomic Analytics software (V.3.0.6.6) was used to visualize, detect and analyze the aberrations deletion and duplication from microarray profiles using Tiff images from data.

### Real-time PCR

Real-time quantitative PCR (qPCR), used to confirm the deletions and duplications detected by array-CGH analysis. Primer were designed for the targeted region for validation using real time PCR analysis. Primers were selected for deleted and copy number gain in the genomic region for CUB and sushi multiple domains 1 (*CSMD1*) gene; *LCE3C*, *HPR*, *and GSTTP2*, genes respectively and an endogenous gene β_2_-microglobulin (*B2M*) and control samples. The reaction was run in a final volume of 10 μl, comprising of 05 μl SYBR-Green qPCR master mix (KAPA Biosystems, USA), 10pmol of each primer and 20 ng genomic DNA. All the deletion and duplications was conformed in triplicate for each sample in a 96 well plate which includes control samples, target genes, reference gene and non-template control for each gene. Each run was analyzed with StepOnePlus™ Real-Time PCR Systems (StepOnePlus™ Real-Time PCR, Applied Biosystems, Canada). The Ct is the threshold cycle at which the fluorescence curve reaches an arbitrary threshold; ΔCt is the difference between the Ct of the target gene and that of the reference gene; ΔΔCt is the ΔCt value of the patients obtained by dividing the ΔCt value of the patient with ΔCt value of the control sample. The *T*-test with significant P-value <0.05 was performed to find out statistical significance of the predicted copy number alterations. The sequence of the primers used in the validation studies are added in Table 1.

**Table 1:** List of gene primers used in the present study

S. no	Gene	Primer	Annealing temp.
1	CSMD1 exon 1	Forward: AAGGATGGTTGAGTCCAG	58
2	CSMD1 exon 1	Reverse: GAGATCCAGTCTAGAGAG	58
3	GSTTP2 exon 5	Forward: GAGTTCAAGACCAGCCTG	60
4	GSTTP2 exon 5	Reverse: ATTTCAGGCATGTGCCAC	57
5	LCE3C exon 5	Forward: GTGTACTCCTAAGTGTCC	58
6	LCE3C exon 5	Reverse: CCACAGCAGGAAGAGAC	60
7	HPR exon 1	Forward: GAAGTGAGCTAGTGGCAG	60
8	HPR exon 1	Reverse: CATCTTGGTTGGTCTTGC	58

## Abbreviations

AEDs: Antiepileptic drugs
Array-CGH: Array comparative genomic hybridization
CNS: Central nervous system
CNVs: Copy number variations
CSMD1: CUB and sushi multiple domains 1
DDT: D-dopachrome tautomerase
DDTL: D-dopachrome tautomerase-like
DGV: Database of Genomic Variant
GSTs: Glutathione S-transferase
GSTT1: Glutathione S-transferase theta 1
HPR: Haptoglobin-related protein
ID: Intellectual disability
LCE: Late comified envelope
qPCR: Real-time quantitative PCR
